# Cataphoresis

**Published:** 1896-09

**Authors:** W. W. Moorehead

**Affiliations:** Aledo, Ill.


ARTICLE IV.

### CATAPHORESIS.

BY W. W. MOOREHEAD, D. D. S., ALEDO, ILL.

**Read before the Illinois State Dental Society.**

From the beginning of the history of dentistry to the present day much time and labor have been spent by our profession in endeavoring to relieve our patients from the pain and suffering brought on by the practice of dentistry.

A majority of the people, as a rule, look upon our offices with a greater or less degree of horror, and few indeed are the patients who do not dread to make an appointment with their dentist. To have our patients to know that it is possible to relieve them from the pain and suffering of operations on their teeth would be to have them come to us for appointments with their minds relieved from that fear of being hurt, which I might say has ever been the curse of our profession. And it would also relieve the operator's mind when he is about to fill an appointment with a nervous patient whose teeth are in a hypersensitive condition.

Time and again have we tried to benumb the sensitiveness of the dentine by the use of caustics and desiccants, and some have even resorted to the use of general anæsthetics, but we all know too well the result of these applications. At times we have been successful in treating sensitive dentine, but in the majority of cases we have found that a sharp instrument in the hands of a skillful operator was the best and most reliable obtundent.

The result of these failures have caused the profession to become skeptical about any new method that you allude to for benumbing the dentine.

To have within our power a method by which we can control the sensibility of dentine, and be able to manage some of those uncontrollable cases, is an object that has been sought for by all progressive dentists.

By the use of cataphoresis I think we have a method by which we can control and obtund the most sensitive dentine.

Several of our dental journals have recently contained articles on the subject just referred to; with your permission I shall quote from them: The definition of cataphoresis, as given by Dr. Wm. J. Morton, is this: The movement of fluids, together with the substances they hold in solution, from the positive pole of the electrode conveying a continuous current in tissue toward the negative pole.

It is the flow of fluids from the positive to the negative pole. He also states that the effects of a medicine placed at a positive pole extends only half way. Thus if you place the positive electrode containing cocain on one side of the gum, and the negative electrode on the opposite side, the effect of the cocain will extend only half way; the opposite side will be hypersensitive rather than anæsthetized.

In a recently published edition of Bigelow's International System of Electro-Therapeutics we find an article by Dr. Frederick Peterson claiming that cataphoresis is purely a physical process.

Thus he says: Physically and not physiologically, it is almost certain that the electrical endosmosis is a mechanical and not an electrolytic effect, for two reasons—first, the action will show itself in any single solution whenever a porous partition, such as plaster of Paris, stoneware, etc., is inserted in the path of the current, even when no electrolysis is taking place, that is, no composition; and secondly, because the phenomena may be stated as follows: Whenever a capillary tube containing a liquid sustains a difference of potential between its extremities, liquid is transferred through the tube toward the cathode. The following facts will explain the character of the process in a clearer and more popular form: If two compartments, separated by a membrane, are filled with a fluid, and in each an electrode is placed, there is a streaming of the fluid through the septum in the direction of the galvanic current, that is, from the positive to the negative pole, so that in the course of time there is an increase of fluid in the negative compartment.

Naturally this omosis, as is well known, occurs between two dissimilar fluids, the direction of the ometic current being from the lighter of the denser liquid. But if the anode is placed in the denser liquid, and the cathode in the lighter, this natural ometic current is not only overcome, but reversed.

This streaming movement is analogous to that taking place in the semisolid sarcous substance of muscle when subjected to the constant current, and observed under the microscope, a visible flowing of the contents of the muscular fiber from the positive to the negative pole.

Some may think that the cataphoric action of electricity is a new thing, but such is not the case. The idea of introducing medicines into the system by the action of the galvanic current originated long ago.

Richardson employed it to introduce medicine into the gums for the purpose of extracting teeth in 1859. Beard and Rockwell in their work on medical and surgical electricity refer to several gentlemen who claim to have carried medicines into the system by the use of electricity.

Owing to the extreme sensitiveness of the dentine, an apparatus that can be used at other points cannot be used on living teeth. To use cataphoresis on living sensitive teeth it is absolutely necessary that you have an instrument by which you can at all times have perfect control of the current. We must be able to raise this element from a weak to a strong current by such minute gradations, that it will be almost imperceptible to the patient.

Such an instrument or rheostat is now manufactured by the Electro Therapeutic Company, known as the Wheeler fractional volt selector. With this selector you may use a battery of about thirty cells, or Edison 110 volt street current.

After connecting the selector with the battery, or street current, place a strip of blotting paper which has been saturated with a solution of iodide of potassium on the binding posts on the top of the selector, and the positive pole will give a reddish spot. To this pole attach the platinum pointed or positive electrode, to the other pole attach the negative, or sponge electrode.

The connections having been properly made you are ready to use medicine by the process of cataphoresis.

Always adjust the rubber dam, insulate any metal fillings that may be close to the cavity you wish to operate upon, this may be done by coating them with temporary stopping, or chloro-percha, cut away the walls of the tooth just enough to gain access to the cavity; in which you may now place a pellet of cotton saturated with the obtunding solution you prefer to use; give the patient the negative, or sponge electrode saturated with a weak solution of salt water, and instruct them to hold it in the palm of the hand or on the face, apply the positive or platinum pointed electrode to the cotton in the cavity, and sometimes it is necessary to moisten this during the application of the current.

Begin with a current that is at first imperceptible to the patient, turn on the dial on top of the selector slowly, and at some point between four and eight volts the patient will experience a slight pain, at this point allow the current to run a few minutes and then raise it slowly to the number of volts that may be required to produce the desired anæsthesia. Owing to the difference in the resistance that people of different temperaments offer to the electric current, it is impossible to designate the number of volts that may be required for each case. It is not necessary to use a strong current in cases of exposure of the pulp, or in young patients.

But by prolonging the application, and using a high degree of voltage, we can benumb the dentine deep enough to cut into the pulp without causing the patient to flinch Now that we have reached the number of volts we wish to use, you must not turn off the current suddenly, but gradually, until the dial indicates zero.

The time occupied in producing anæsthesia varies from six to twenty-five minutes. Occasionally you find a patient who is extremely susceptible to the current; for such cases use a low degree of voltage, and a prolonged application.

I will now refer to a few cases from practice :

Case 1. Anterior approximal cavity in left upper central incisor, involving at least one-third of the cutting edge, dentine sensitive.

Treatment: Cataphoresis for fifteen minutes, using a 25 per cent, solution of cocaine, reaching a maximum of twenty volts. Result, absolute freedom from pain when using a bur. As I wished to use a screw post, I made a second application, which occupied about six minutes, with this result: absolute freedom from pain while anchoring the screw.

Case 2. Large approximal cavity in the left superior cuspid, condition of tooth such that I had decided to destroy the pulp.

Treatment: Two applications of cataphoresis, using the solution of guaiacol cocaine "recommended by Dr. William J. Morton," for twenty-two minutes, reaching a maximum of eighteen volts in each application. Result, painless removal of pulp and the root and crown were filled immediately. I also wish to state that there was no inflammation or soreness after this operation.

Case 3. Approximal cavity in left upper lateral incisor, patient very nervous, dentine hypersensitive.

Treatment: Cataphoresis for twenty minutes with guaiacol cocaine, reaching a maximum of twelve volts. Result, almost entire freedom from pain, the patient insisted that I was not cutting in the sensitive part of the tooth, even when I was using a bur in the cervical portion of the cavity. By the use of a 25 per cent aqueous solution of pyrozone, discolored teeth may be easily and thoroughly bleached. To prepare this aqueous solution for cataphoresis, McKesson & Robbins recommended that you take of 25 per cent. etheral pyrozone two parts, water one part, evaporate the ether, then add a small quantity of salt, "1 or 2 per cent.," and it is ready for use. Before attempting to bleach a tooth it is necessary that the apex of the root be filled with gutta-percha, or some non-conducting material to prevent the current from carrying the solution through the tooth. Etheral solution of pyrozone cannot be used for bleaching by the process of cataphoresis because they are non-conductors. Much stronger currents may be used in bleaching, than we would use in benumbing the dentine.

The instruments necessary to introduce medicines by the process of cataphoresis may be obtained from the Electro-Therapeutic Company, 32 East Twenty-third Street, New York, or from the Galvano-Faradic Manufacturing Company, of the same city.

The advantage of cataphoresis over other methods of introducing medicines is that you can carry your solutions not only into soft tissues, but through solid dentine, Many medicinal solutions are non-conductors, and to use such solutions by the cataphoretic process you must add to them a substance that will cause the same to become electrolyte. Thus Dr. Morton adds 1 minim sulphuric acid to about two drachms guaiacol, and to pyrozone we would add salt or soda, say about 1 per cent.

It is not necessary that we should be electricians in order to be successful with cataphoresis, but it is absolutely necessary that the operator should take enough time to make the application in a careful and thorough manner. To all such operators I would recommend cataphoresis with the assurance that they and their patients will be benefited many times thereby.

Usually one fifteen minute application will permit the entire preparation of the cavity, but if much cutting is necessary it is advisable to make a second application; some may think this process takes too much time; to such I would say, "Let cataphoresis alone."

While I do not claim originality for this method of producing anæsthesia, yet I do assert that it has enabled me to assure my nervous patients that it is possible to fill their sensitive teeth without pain.—*The Dental Review.*

